# Bend it like Beckham! The Ethics of Genetically Testing Children for Athletic Potential

**DOI:** 10.1080/17511321.2013.780183

**Published:** 2013-04-04

**Authors:** Silvia Camporesi

**Affiliations:** Centre for the Humanities and Health, 5th floor, East Wing, King’s College, London, WC2R 2LS, UK

**Keywords:** DTC genetic tests, children, sports, talent, pre-professionalization, open future, autonomy, practice

## Abstract

The recent boom of direct-to-consumer (DTC) genetic tests, aimed at measuring children’s athletic potential, is the latest wave in the ‘pre-professionalization’ of children that has characterized, especially but not exclusively, the USA in the last 15 years or so. In this paper, I analyse the use of DTC genetic tests, sometimes coupled with more traditional methods of ‘talent scouting’, to assess a child’s predisposition to athletic performance. I first discuss the scientific evidence at the basis of these tests, and the parental decision in terms of education, and of investing in the children’s future, taken on the basis of the results of the tests. I then discuss how these parental practices impact on the children’s right to an open future, and on their developing sense of autonomy. I also consider the meaning and role of sports in childhood, and conclude that the use of DTC genetic tests to measure children’s athletic potential should be seen as a ‘wake up’ call for other problematic parental attitudes aimed at scouting and developing children’s talent.

## 1 Introduction

‘Bend it like Beckham’ is the title of a 2002 Golden Globe-nominated movie by British director of Indian origin Gurinder Chadha. The movie features a young talented girl named Jess (Parminder Nagra), who dreams of becoming a professional soccer player, but she is not allowed by her parents to join a team because of her double identity as female, and Indian. After much family fighting, Jess finally escapes traditional conceptions of Indian femaleness to flee to the USA where she is able to play with a college scholarship at Santa Clara University, CA. It seems to me that the title of the movie encapsulates well the parental desires and motivations underlying the recent boom (especially in the USA) of direct-to-consumer (DTC) genetic tests aimed at measuring athletic potential (Chang 2009; Macur 2008; Stein 2011). Parents aim to gain an early advantage (a ‘head start’) which would allow their children to turn already at an early age into professional athletes, and continue on a hoped-for chain of events from college scholarship to success, fame and money. What, if anything, is problematic with this?

I have argued elsewhere that parents should not be allowed to resort to pre-implantation genetic diagnosis (PGD) to choose to have deaf children like themselves, on the basis of the rights of the children to a/an (sufficiently) open future and on the limits of parental reproductive freedom (Camporesi 2010). In this paper, I consider another kind of intervention that may at first sight appear much less ‘radical’ than intervening at the level of PGD to mold children’s futures. This would be the use of genetic tests, sometimes coupled with talent scout camps, to assess the child’s predisposition to athletic performance. I first discuss the scientific evidence at the basis of these tests, and the parental decision in terms of education and investing in the children’s future taken on the basis of the results of those tests. I then discuss how these parental practices impact on the children’s right to an open future (ROF), and on their developing sense of autonomy, and consider the meaning and role of sports in childhood.

## 2 Genetic Tests for Athletic Performance

In the USA, there are at least seven companies that sell DTC genetic tests for sports performance or related traits, probably more (Roth 2012). The prices for these tests are quite affordable, thanks to the constant lowering of the costs of genome sequencing, and vary from approximately $80 to $200. Among these companies feature ‘Sports X factor’, ‘Atlas Sport Genetics’, ‘Athleticode’, ‘Geneffect’, and ‘Warrior roots’. Since such data is proprietary, it is not clear exactly how many parents and coaches are using these tests, but based on the number of companies thriving on the market, we can speculate that hundreds of parents and coaches are using them (Brooks and Tarini 2011). Note that since these tests are available on the Internet, the market is not limited only to the USA, but potential customers in the UK, Europe or rest of the world could order the test online, and only have to pay higher shipping expenses for the test-kit.

Sometimes, these tests are coupled with more ‘traditional’ methods for talent scouting, as a story published by the CNN shows (Chang 2009). The story tells of a camp set up in Chongqing, a major city in south-west China. In the so-called ‘Children’s Palace’, about 30 children between the ages of 3 and 12 were selected to participate in an innovative programme that combined traditional methods of talent scouting with genetic testing with the goal of giving Chinese children ‘an effective, scientific plan [of development] at an early age’ as put by Director Zhao Mingyou. The Chinese Government then takes care of implementing this ‘effective, scientific plan’. I will not consider in this paper the role of the government in education, as I want to restrict my analysis to the role of parents; but it is interesting to note that talent scout camps like the one in Chongqing are a possible future in the West.

### 2.1 What are these Tests Testing for, and What is their Predictive Value?

Most companies test for a panel of what they call ‘performance enhancing polymorphisms’ (PEP), a few only for one. All of them test for the alpha-actinin 3 (ACTN3) polymorphism, which I will describe in detail below. Although many genes and gene sequence variants have been tentatively associated with performance-related traits, few if any have risen to a level that would be called conclusive. As Roth (2012) recently pointed out: ‘This is not a judgment against the existing science, but rather a recognition of the *infancy* of the field of exercise and sports performance genomics’. Not only is the field of genetics of sports performance in its infancy, but the DTC genetic tests take data obtained in one pool of subjects (i.e. elite athletes) and apply them to a substantially different one (i.e. children, teenagers) in what Eynon et al. (2011) refers to as the problem of ‘externality’.

As an example, I will focus on the test for ACTN3 polymorphism, which has the most robust scientific basis: ACTN3 was the first PEP to be demonstrated to have an association with skeleto-muscle formation and function, and is offered by all the companies available on the market. Therefore, any criticisms directed against this test will be valid also—even more so—against the other tests.

In 2003, Yang et al. found a significantly higher frequency of the functional 477R genotype in the ACTN3 gene (where R stands in place of an arginine ‘R’ rather than a stop codon) in both male and female elite sprinters (Yang et al. 2003). Alfa-actinin is an actin-binding protein, where actin is an integral component of the protein superstructure that generates contractile force within muscle fibers. Polymorphism in ACTN3 are thought to contribute to the heritability of fiber-type distribution in muscle, where the Type I are slow-twitch fibres that metabolise aerobically and are used in endurance races, while Type II are fast-twitch fibres that metabolise anaerobically, and are used in sprints (Ostrander, Huson, and Ostrander 2009).

The test for ‘ACTN3 Sports Gene’ is sold as a genetic ‘Power/Speed performance test’, and as we can read on the website of Atlas Sports Genetics (one of the companies that offer the test) with the aim to give ‘parents and coaches early information on their child’s genetic predisposition for success in team or individual speed/power or endurance sports’ (http://www.atlasgene.com/). We can also read that the results of the tests will be ‘valuable in outlining training and conditioning programs necessary for athletic and sport development’ (ibid.).

The patent exploitation of the infancy of this field of research by the companies has been referred to by Caulfield (2011) as ‘scienceploitation’, or the ‘exploitation of legitimate fields of science and, too often, patients and the general public, for profit and personal gain’. A case in point for scienceploitation: the tests for ACTN3 variant claim to assess the predisposition to athletic ability and prowess, while the ACTN3 gene accounts for only 2% of total variance in muscle performance (Eynon et al. 2011). The rest of the variation is determined by a wide range of genetic and environmental factors, most of which (particularly the genetic factors) are very poorly understood.

In addition, as pointed out by McArthur (2008) (note that McArthur is one of the authors who demonstrated the higher frequency of the ACTN3 polymorphism in elite sprinters), the fact that there is a higher frequency of ACTN3 polymorphism in elite sprinters does not mean that the test is actually predictive of athletic performance, as muscle performance (of which the ACTN3 variation accounts for only 2%) clearly does not equate with athletic prowess, notwithstanding what the companies are claiming.

Finally, these tests pose a potential problem with false negatives, as the parents will act upon the results of these tests and the claims made by the companies and actively discourage their children from a particular kind of sports for which they allegedly do not have a genetic predisposition. For example, the company Geneffect frames the results of the ACTN3 test in terms of ‘genetic advantage’ for ‘Sprint, Power & Strength Sports’ for a RR genotype, for ‘Endurance Sports’ for a XX genotype and for a ‘Mixed Pattern Sports’ (equivalent for ‘any other sport’) for a heterozygous genotype (http://www.geneffect.com/actn3/en/results.html).

Following a classification by Caulfield (2011) of DTC genetic tests into the three partially overlapping categories of: (a) the clearly preposterous; (b) the marginally pertinent; and (c) the vaguely predictive, we could say that, in a charitable interpretation, DTC genetic tests offered by companies such as Atlas Sports Genetics, Sports X Factor, or Geneffect would be classified as marginally pertinent, while in a less charitable interpretation, they could be classified as clearly preposterous.

Note that I do not think that the inability of DTC genetic tests to predict children’s athletic performance is a matter of contingency in science or the infancy of the field. I am persuaded that DTC genetic tests will never be able to predict something as complex as athletic talent, even if the association were replicated in larger population samples and, therefore, strengthened. I am not interested in discussing the ethical implications of ‘GATTACA-like’ science fiction scenarios where genetic tests are able to predict intelligence or other complex character traits. I think that athletic excellence is simply too complex a trait to be possibly pinned down to single or even multiple genetic associations in a deterministic fashion. This said, it is a matter of fact that information framed in terms of genetic knowledge is charged with an extra ‘authoritative aura’ that seems to be intrinsic in the G, A, T and C bases of the deoxyribonucleic acid. It is also a matter of fact that these companies market their tests, and that at least some parents accept their results, as if they were deterministic in nature, and as if they were really able to predict the talent of their children. Therefore, parents act upon these tests and make decisions on the basis of the results that involve investing in their children’s future. By doing so, these tests acquire a causal significance in the lives of these children.

The rest of this paper will analyse the ethical permissibility of the parental practices independent of the above criticism on the scientific validity of the claim. As I see them, DTC genetic tests are a new instance of a wave of criticizable parental approaches to childrearing, and they should function as a ‘wake up call’—borrowing an expression from Davis (2009)—for other current practices of directive childrearing that deserve a closer scrutiny, and critical analysis.

## 3 Parents Scouting their Children’s Talents

Brad Marston, father of nine-year-old prospective soccer player Elizabeth, is a satisfied customer and a testimonial for Atlas Sport Genetics. His story can be read on the company website (http://www.atlasgene.com/index.php?do=testimonial):

Atlas Sports Genetics testing was very informative and the process was quite simple. Although my daughter is only 9 she now knows that she has the ‘Sprint, Power, & Strength advantage’ which we can use to market her athletic career and hopefully a wonderful scholarship from this process.

Brad Marston does not represent the emergence of a new kind of parent. On the contrary, he represents a new instance of an old kind of parent: parents who employ all kinds of methods to encourage or steer their children towards a life of athletic, musical or other professionalism. Parents have always done so: from submitting their children to heavy training schedules, to intensive summer camps, to hiring private teachers and tutors, and so on and so forth. While these practices are occasionally subjected to criticisms for their strictness, it is generally accepted that it is permissible within the parental role to steer children even aggressively in a particular direction. These kinds of attitudes can be reinforced by the consequences, i.e. if the child later in life is actually successful in her sport or music activities, her success seems to confirm the ‘rightness’ of the childrearing parental behaviour, in a kind of retroactive approval, or consent that takes the form of: ‘See, it was worth it’ or ‘I was right in the end’, etc.

DTC genetic tests aimed at measuring the athletic potential of a child can be seen as the latest tool available to parents to steer their children’s future, and their investments, with the expectation that their efforts will be—quite literally—‘paid off’. Is it justifiable for parents to do so?

Feinberg (1980) has defined the child’s ROF as a ‘vague formula that describes the form of the particular rights in question but not their content’. The rights in question are ‘rights in trust’, or anticipatory autonomy rights: they look like adult autonomy rights, except that the child cannot exercise her choice until later. The violation means that when the child is an autonomous adult, certain key options will be already closed to her, undermining her capacity for self-determination (which Feinberg sees as a necessary condition for self-fulfillment in life). As already noted by Dixon (2007), Mills (2003) objects to Feinberg and argues that not only is it impossible to actually have an open future due to the finitude of our lives, and to the inevitable closure of possibilities that takes place every time we make a choice, but also that it would not even be a desirable option. For Mills, parental approaches that aim to leave their children with an open future consequently expose them to a frenetic ‘smorgasbord’ of activities, and end up being detrimental to a vision of more profound and authentic experiences of the life of a child. This more profound vision would encompass also a meaningful ‘idleness’, a time for play that is not necessarily goal-directed (success, fame), and that privileges the child *hic et nunc* vs. the successful and possibly burnt-out adult that the child will grow into.

I find the analysis by Mills very compelling: it seems true to me that some parents are constantly projecting into the future of their children, and do not give a proper value to the present child that they have in front of them. What was once ‘free time’ from school and homework has become time devoted to activities x, y, z, which by virtue of being activities that are goal-directed (talent-scout, talent-development directed) lose their value of free time, of idle time that is supposed to act as a counterbalance to the already many compulsory activities that a child has to undertake early on in her life. But, this is only part of the story, as Lotz (2006) has correctly pointed out. Lotz, while recognizing the validity of some of the worries raised by Mills against the smorgasbord approach adopted by some parents, shows that such criticisms are not really directed to Feinberg’s, but to current trends of childrearing and educating driven by excessively competitive parents. In other words, striving to protect a child’s ROF does not commit parents to the problematic ‘smorgasbord attitudes’ described by Mills. Indeed, if we look at the original source, we can see that Lotz is right in her analysis, and that Feinberg is well aware of the inevitable narrowing of options in parenting:

[…]Simply by living their own lives as they choose, the parents will be forming an environment around the child that will tend to shape his budding loyalties and habits. (Feinberg 1980)

This narrowing of possible futures is inevitable in the practice of parenting and especially so in the case of talented children, but does not necessarily violate the child’s ROF, provided that the child’s input is taken, whenever possible, into consideration. How is that possible in practice?

## 4 What it Means to be a Child and Discretionary Domains of Autonomy

Archard (2004) argues that parents cannot avoid (nor would it be desirable if they tried) forming their children’s characters to some extent. He writes: ‘It would be a caricature of ideal liberal parents to imagine them zealously striving to avoid the creation of any particular personality in their children’ (Archard 2004, 56–57). Archard acknowledges that the choices made by parents concerning their children’s rearing and education have an ‘opportunity’ cost for their children, namely the absence of some other upbringing, but this is unavoidable. Moreover, self-determination of the child is not the only value to take into account when evaluating upbringing. A good upbringing should realize the child’s talents, and these may be realized sometimes only to the detriment of self-determination, and, therefore, of the child’s open future. Talented children are particularly difficult cases, as the nurturing of a precocious musical or sport talent may lead to a successful adult (concert soloist, Wimbledon tennis player, etc.), but that will have been achieved at the expenses of other skills (possibly, all other skills except that particular one which was nurtured) and of the person’s self-determination. How is it possible to preserve the child’s budding sense of self-determination, while also nurturing her talents? As said above, Feinberg’s analysis of the children’s ROF is that of a ‘right in trust’, i.e. a right to be saved for the child until she becomes an adult. I will move now to the analysis of what it means for a child to become an adult, and what implications this process has on the development of the child’s autonomy.

Schapiro (1999) addresses a very important but fairly neglected question: what is a child, such that it could be appropriate to treat a person like one? In tackling this question, Schapiro is addressing also the following two related questions: (a) When is a parent justified in preventing a child from acting according to her own will? and (b) When is a child entitled to make her own choices?

Schapiro draws a parallel between children being provisional, passive members of the political community with children also being provisional, passive members of the ethical community. Their status of passivity is provisional because of their liminal and ever-changing status, and their condition of moving towards adulthood. Indeed, as children at different stages of development differ from one another in the extent to which they have hegemony over themselves, they also differ in the relative status of their passivity as members of the ethical community. Children gain access to the ethical community once they gain autonomy and sovereignty, as put by Schapiro, by developing increasingly broader ‘domains of discretion’. Once they have achieved sovereignty over some domain of discretion (e.g. being able to eat without being fed, being able to get dressed alone and so on and so forth), parental obligation would require that children be left to decide and exercise autonomy over that domain. In this way, writes Schapiro, the child is forced to come up with provisional principles of deliberation that function then as starting points, as anchors, for ‘ever widening domains of discretion’. Along similar lines, Feinberg also writes: ‘The child can [and should, I would add] contribute towards the making of his own self and circumstances in ever-increasing degree’ (Feinberg 1980, 736). This contribution to her own self-determination entails, I think, also exercising her autonomy over the sport she (the child) wants to play, or does not want to play.

The parental practices exemplified by the use of DTC genetic tests to provide children with a ‘head start’ in life are deeply problematic because—as put by Wall (2010)—they interpret children ‘only through the lens of what they are not yet, namely adults’ (Wall 2010, 144) and do not take into account the *in fieri* moral agency of the child. Borrowing again from Wall, while it may seem an obvious goal that the main purpose of a family and of parenting is ‘helping children to grow up into adults’, this practice ‘obscures the ethical sense in which children are diverse and other moral agents in and of themselves’ (Wall 2010, 144). Children should expect from their parents to be equipped with a range of broad skills that will enable them to make autonomous decisions and choose their path in life. On the converse, being equipped with very specific skills (like playing pre-professionally soccer, football, volleyball and so on) very early in life and having a life plan spelled out for them would constitute a brake on their development, and relegate them to being passive receivers of education. In addition, by depriving children of the possibility to exercise their budding self-determination, it relegates them to being passive members of the moral community. The possession of a ‘life plan’ early on in life has been defined by Slote as both ‘unnatural’ and ‘unfortunate’ (Slote 1989, 40–41). ‘Life-planfulness’ as a character-trait is seen by Slote as a virtue with a temporal aspect, i.e. a ‘positively good thing in individuals mature enough […] to decide upon a career or profession’, but becomes an obstacle for development in children, a ‘brake’ to the existence itself of their, although limited, autonomic domains of discretion.

What about children with talents? Slote recognizes that an early start can be necessary for the fulfillment of that talent, as he writes (though he writes it in a footnote, so he must not have considered their case too important):

All this *[considering a life plan a bad thing in children]* is consistent with allowing that those who make premature life plans concerning careers are sometimes very successful in those careers. But such premature choices are typically the result of parental pressure, and those who yield to, and succeed under, such pressure can hardly help being emotionally scarred by it as well. (ibid., 47)

An example that comes to mind is Andre Agassi, the American Hall of Fame tennis player whose father allegedly tied a tennis racket to Andre’s hand when he was only three years old, and obliged him to hit tennis ball after tennis ball that were being literally spit out by a dragon like-machine built by his father specifically for that purpose (Agassi 2010). In his autobiography, Andre Agassi is very resentful towards his father and the education he was submitted to: even though Andre grew up to be one of the world’s most famous tennis players, he achieved that at the expenses of all other skills, including basic school education (Note that both Andre’s older siblings, being also talented children in tennis, were submitted to a similar education, but never made it to a professional career).

As said above, talented children are tough cases exactly because they embody the tension between nurturing talent and the self-determination capacity of the child, both of which are considered two duties of a good parent. Indeed, it can be plausible to argue that the particular kind of precocious and ‘absolutist’ upbringing necessary to nurture the child’s talent was the only possible way to achieve success in a domain where early training and early gaining of a competitive advantage is essential. It seems, therefore, that parents must strive both to realize the child’s particular talents and to safeguard her ‘open future’, walking along what we could call a kind of ‘imaginary fence’ and trying to keep a difficult balance between the good of this particular child (realising the present) and the good of the adult that the child will grow up to be (the future). The tension between these two goals will be exacerbated when these goals are defined in maximizing terms, i.e. the Andre Agassi or the concert soloist at Royal Albert Hall and so on and so forth. In the next paragraph, I will consider what role and significance sport should play in childhood.

## 5 The Meaning and Significance of Sport in the Child

As noted by McNamee et al. (2009), if it is appropriate to say that the research field of ‘sports ethics’ is in its infancy, then it could be said that the research field of ‘sports medicine ethics’ is neonatal. The analysis of genetic tests for athletic performance falls within this ‘neonatal’ realm. Note also that the comment by Roth (2012) on the infancy of exercise and sports genomics falls along the same lines. Within the infant field of sports ethics, Mathias (2004) has written a rare and well-argued review of its history. Mathias defines both (elite) sports and medicine as goal-directed activities: the former as having ‘victory’ as one of its goals, the latter ‘health’. Both ‘victory’ and ‘health’ are regarded as goods by the subjects involved in the activities, and these goals may very well be, and often are, in contrast in elite sports (e.g. return to play decisions). As noted by Mathias, ‘the history of ethics in sports medicine has been driven by the general tension between the demands of sport and the demands of health’ (Mathias 2004, 196), and, therefore, we should ‘not be surprised to find in the field where they come together, sports medicine, signs of this tension occur in the form of persistent ethical problems’ (ibid.). It needs to be noted though that the aim of sports in children need not necessarily be ‘victory’, quite on the contrary.

What is the role played by sports in children? I argue that it should not be a goal-directed activity (directed to victory), differently from what it is for the athlete who is engaged in a professional, elite sport. Sport in children could instead be understood as a ‘practice’, defined by MacIntyre (1984) as a coherent and complex form of socially established cooperative human activity through which goods internal to that form of activity are realized in the course of trying to achieve those standards of excellence which are appropriate to, and partially definitive of, that form of activity (MacIntyre 1984, 186). In this sense, sport as a practice in childhood becomes defined both by goods internal to the practice (e.g. to stay healthy, enjoy the company of friends, enjoy the discovery of the possibilities of one’s own body, learn how to relate with a team, learn the importance of rules, etc.) and by the standards of excellence of the practice (i .e. nurturing and developing talent). Going back to Slote and his analysis of the temporality of virtues, we could also say that some goods are inherent/intrinsic to childhood (including engaging in a sport as a practice, and not as in a competitive profession directed to victory) and should be preserved exactly for that reason.

Therefore, parents could, and should, expose children to a variety of sport activities (and other non sport-related activities) compatible with their financial situations, and their own preferences and ways of living. In this sense, I think that parents could and indeed should be free to live ‘their own lives as they choose’, as put by Feinberg (quoted above), as long as they ‘do not isolate children intentionally from other ways of life, and make sure that children learn of the variety of ways of life’ (Lotz 2006, 541). If, for instance, a set of parents love hiking, then they will expose their children to outdoor sports, while other kinds of parents may expose their children to more indoor activities, like music, or team sports that are played indoors. This seems to be perfectly reasonable, as long as the other option is not completely cut off from the child’s horizon. What seems unreasonable is to expose the child to one and only one sport, and actively discourage any other.

## 6 Conclusions

In this paper, I have analysed a new tool that parents have to steer their children’s education and develop their talents: DTC genetic tests for athletic potential. After analysing their scientific and medical basis of their predictive value for the most widespread test (ACTN3), I showed that in the best possible scenario they are only marginally pertinent, with gross misrepresentation of their predictive value in the marketing claims of the companies. I have framed the issue as a new instance of the debate of the Feinbergian children’s ROF, but complemented it with arguments on what it means to be a child (in an ethical way), the time preference of certain character traits and the meaning of sports in children as a ‘practice’. In particular, I argued that Schapiro’s analysis of the child’s autonomy in terms of ‘domains of discretion’, combined with Slote’s temporality of the character trait of ‘life-planfulness’, can be useful lenses to analyse parental approaches of childrearing, and complement classical arguments of the child’s ROF.

Within the children’s domain of discretion falls the choice of which sport (if any) to play, which I argue should be free from talent-related reasons (as there are many other values in sports for a child) and from any life plan that the parents may want to impose on their children. Parental violation of the child’s domain of discretion is not only a violation of the child’s ROF, but also a violation of the child’s actual preferences. In this sense, I take into account Mills’ concerns and value the autonomy of the present child, as much as the autonomy of the adult that the child will grow into.

In the end, I recognize the existence of an unavoidable tension between the goal of maximizing children’s talents and nurturing their self-determination, but I am inclined to view the latter as more important. Nonetheless, I recognize the impossibility and non-desirability of non-directiveness in childrearing, and I find the criticism by Mills of ‘smorgasbord’ parental attitudes quite appropriate and resonant with current Western trends of parenting.

These arguments form my two-pronged rationale to object to the parental use of DTC genetic tests to (supposedly) measure their children’s athletic potential, and to steer their education towards an early start to a professional sports career. I am aware here of two possible challenges to my arguments, namely that my dismissal of the ‘success stories’ argument derives from not being myself one of those success stories; and that I am not qualified in my critical analysis of parents’ childrearing practices being not yet a parent myself. These are true. Points taken. But, as for the first point, I would like to underline that only a very small per-cent of the totality of children who underwent an ‘absolutist’ upbringing devoted only to nurturing one particular talent become stories of success, while all of them are raised to the detriment of a complete development of the person, of her self-determination, and possibly of all other skills, and with ‘no small emotional scars’, as put by Feinberg. As for the second point, I will be happy to take on the challenge again in—maybe—a few years.
